# Editorial: Break the mental health stigma: eating disorders

**DOI:** 10.3389/fpsyt.2024.1416250

**Published:** 2024-05-15

**Authors:** Faisal A. Nawaz, Hatice Kurdak, Antonios Dakanalis, Marios Argyrides, Rahul Kashyap

**Affiliations:** ^1^ Al Amal Psychiatric Hospital, Emirates Health Services, Dubai, United Arab Emirates; ^2^ Department of Family Medicine, Faculty of Medicine, Cukurova University, Adana, Türkiye; ^3^ Department of Mental Health and Addiction, Fondazione Scientific Institute for Research, Hospitalization and Healthcare (IRCCS) San Gerardo dei Tintori, Monza, Italy; ^4^ Department of Medicine and Surgery, University of Milan Bicocca, Milan, Italy; ^5^ Department of Psychology, Neapolis University Pafos, Pafos, Cyprus; ^6^ Department of Research, WellSpan Health, York, PA, United States; ^7^ Department of Anesthesiology and Critical Care Medicine, Mayo Clinic, Rochester, MN, United States

**Keywords:** mental health, stigma, eating disorder, bulimia nervosa, anorexia/bulimia

## Introduction

Eating disorders (EDs) represent a group of mental health disorders that significantly affect both the physical and psychological well-being of an individual (1). According to the fifth edition of the Diagnostic and Statistical Manual of Mental Disorders (DSM-5), there are five main categories of eating disorders: Anorexia Nervosa (AN), Bulimia Nervosa (BN), Binge-Eating Disorder (BED), Other Specified Feeding or Eating Disorders (OSFED), and Unspecified Feeding or Eating Disorders. The prevalence of EDs among adolescents is ranked third among other chronic illnesses, second to only obesity and asthma (1). Stigma related to EDs is a growing challenge that impacts patient access to mental health care on a global scale. In a cross-sectional study conducted by Griffiths et al. studying 317 individuals with eating disorders, stigmatization has been correlated with an exacerbation of psychopathological symptoms, an extended duration of the disorder, diminished self-esteem, and an increased internalized stigma related to seeking mental health support (2). Till date, there is limited literature and resources allocated to tackling the stigma related to this topic. The aim of this editorial is to shed light on the valuable contributions of articles published in the Research Topic titled “*Breaking the mental health stigma: eating disorders*”. This article also highlights the future direction and recommendations for improving the quality of care and research on eating disorders.

## Current research

Kurdak et al. conducted a bibliometric analysis exploring research trends and patterns related to EDs, stigma and primary care. Research publications on this topic were most active in United States, followed by the United Kingdom, Australia, and Canada. The topic analysis on this subject included 541 published articles and indicated a growing research trend. Nonetheless, there were a limited number of research studies addressing the stigma surrounding EDs, which was a noted concern. The study further emphasized the role of primary care in supporting early screening measures for patients with EDs, the need for specialized training on this topic, and the importance of collaboration and expanding primary care-related research in this field.

Özbaran et al. conducted a retrospective study to observe outcomes of a follow-up model for pediatric EDs at an outpatient clinic. Upon confirming the ED diagnosis of 37 patients aged 12–17 years, clinical variables analyzed included Body mass index (BMI), Clinic Global Impression (CGI) scale scores, duration of follow-up, number of interviews and other scale scores (The Turgay Attention Deficit Hyperactivity Disorder Scale and the Autism Spectrum Screening Questionnaire Scale). It was found that the BMI at the last clinical interview increased with increasing follow-up time (r = 0.561, p < 0.01) and number of interviews (r = 0.348, p = 0.038).

Another study conducted by Özbaran et al. aimed to assess the psychosocial and clinical impact on patients with AN during the Coronavirus Disease 2019 pandemic. This cross-sectional study among 35 patients revealed that the pandemic had adverse effects on the AN patients and disrupted their daily lifestyle, alongside a higher rate of comorbidities. It was also noted that during the pandemic period compared to the pre-pandemic period, there was a significant increase in obsessions, exercise patterns and screen time using technology among these patients. At the same time, quality of education and perception of learning decreased during this time.

Huang et al. focused on the validation of the Sociocultural Attitudes Towards Appearance Questionnaire-4 Revised (SATAQ-4R) in the Chinese adolescent population. The sample included 344 females and 335 male high school students that were given the SATAQ-4R for completion. The internal consistency of both gender-based scaled was rated as good, with convergent validity as acceptable, while significant correlations were noted in the female subscale. The study also clarified the structure of this tool for adolescents aged 15–17 years old, which was not previously validated in other studies.

The perspective article by Nawaz et al. shared insights on the potential of social media among adolescents with EDs as a medium for negative outcomes as well as its use as a for positive community building initiatives in this population. The article highlighted key challenges in social media content regulation, misinformation, and lack of evidence-based resources. Recommendations for change include online awareness campaigns, increased research towards social media user behavior and impact in this population, and anti-bullying initiatives to promote a safe patient culture online.

## Future direction and conclusion

Several key insights were gained from this research series, which paves the way for future research and clinical innovation. Future studies should focus on the cross-cultural adaptation and validation of EDs assessment tools in these regions. This will ensure the accurate identification and understanding of EDs within diverse populations, fostering inclusive and effective interventions. Primary care settings remain underutilized in the early detection of EDs. Research should explore models for integrating EDs screening and prevention strategies within primary care. This could involve training primary care providers in recognizing EDs symptoms and establishing referral pathways to specialized care, thereby facilitating timely intervention. To enhance the generalizability of research findings, future studies must employ larger and more diverse sample sizes of patients with EDs. Furthermore, social media holds potential as a tool for challenging the EDs stigma. Research should investigate how these platforms can be leveraged to foster positive narratives around EDs, support community building, and provide accessible resources for individuals affected by EDs.

In conclusion, the Research Topic “*Breaking the stigma: eating disorders*” brings forward a successful series of articles that highlight the need for a multifaceted research approach in tackling the stigma against EDs. Concentrating on research into eating disorders is pivotal for enhancing health outcomes by identifying effective treatments and intervention strategies, fostering a global culture of safety and openness, while encouraging patients to engage in conversations as vital stakeholders in the healthcare field.

## Author contributions

FN: Conceptualization, Investigation, Methodology, Project administration, Resources, Writing – original draft, Writing – review & editing. HK: Conceptualization, Methodology, Project administration, Supervision, Writing – original draft, Writing – review & editing. AD: Conceptualization, Methodology, Project administration, Supervision, Writing – original draft, Writing – review & editing. MA: Conceptualization, Methodology, Project administration, Supervision, Writing – original draft, Writing – review & editing. RK: Conceptualization, Investigation, Methodology, Project administration, Resources, Supervision, Writing – original draft, Writing – review & editing.
